# Structural Aspects of the O‐glycosylation Linkage in Glycopeptides via MD Simulations and Comparison with NMR Experiments

**DOI:** 10.1002/cphc.201900079

**Published:** 2019-05-06

**Authors:** Aysegül Turupcu, Matthias Diem, Lorna J. Smith, Chris Oostenbrink

**Affiliations:** ^1^ Department of Material Sciences and Process Engineering, Institute of Molecular Modeling and Simulation University of Natural Resources and Life Sciences Vienna Vienna Austria; ^2^ Department of Chemistry University of Oxford Oxford U.K.

**Keywords:** Glycosylation, Molecular dynamics, Glycopeptides, NMR spectroscopy

## Abstract

A powerful conformational searching and enhanced sampling simulation method, and unbiased molecular dynamics simulations have been used along with NMR spectroscopic observables to provide a detailed structural view of O‐glycosylation. For four model systems, the force‐field parameters can accurately predict experimental NMR observables (J couplings and NOE's). This enables us to derive conclusions based on the generated ensembles, in which O‐glycosylation affects the peptide backbone conformation by forcing it towards to an extended conformation. An exception is described for *β*‐GalNAc‐Thr where the *α* content is increased and stabilized via hydrogen bonding between the sugar and the peptide backbone, which was not observed in the rest of the studied systems. These observations might offer an explanation for the evolutionary preference of *α*‐linked GalNAc glycosylation instead of a *β* link.

## 1 Introduction

Glycosylation is a co‐ and posttranslational modification (PTM) of proteins which is widely observed. It is the most diverse form of PTM, since different glycosidases and glycosyltransferases within the ER and Golgi apparatus give rise to different patterns of protein glycosylation in each cell line. This variety contributes to the production of glycoproteins with different and specific functions. It has been shown that glycans have important biological roles; such as correct folding of proteins, recognition events important for development, differentiation of a particular cell, tissue, or organism.^1^

N‐ and O‐glycosylation are the two most common types of glycosylation where glycans are attached to proteins via either the nitrogen of Asn (N‐linked) or the oxygen of Ser/Thr (O‐linked). In some limited cases O‐glycans can be attached to modified hydroxyproline and hydroxylysine and in a very rare event, O‐glycosidic linkage of *α*‐glucose to tyrosine is observed in glycogen containing eukaryotic cells.^2^, ^3^, ^4^ The consensus sequence for N‐glycosylation is Asn‐X‐Thr/Ser where X is any amino acid different than Proline. On the other hand, O‐glycosylation does not have a defined sequence motif in the protein. The most common O‐glycosylation type in mammals is the mucin‐type glycan or O‐GalNAc glycans where the first carbohydrate residue is a conserved N‐acetylgalactosamine (GalNAc) which is covalently *α*‐linked to the side chain hydroxyl substituent of serine or threonine.^5^ They are found on many secreted and membrane‐bound glycoproteins in eukaryotes. There are also non‐mucin types of O‐linked sugars in mammalian cells including *α*‐linked fucose and mannose; *β*‐linked xylose and N‐acetylglucosamine (GlcNAc), and *α* or *β*‐linked galactose and glucose. Examples of these unusual non‐mucin O‐linked sugars are O‐fucose found in epidermal growth factor (EGF) domains; O‐GlcNAc on cytosolic and nuclear proteins and O‐mannose in bovine peripheral nerve *α*‐dystroglycan.^6^, ^7^

The importance of O‐glycosylation is documented through its effect on the properties of the proteins such as increasing the viscosity in mucins or increasing the solubility of the proteins in venoms.^6^, ^8^, ^9^, ^10^ In addition to its direct effect on the protein, O‐glycosylation also has a notable role in protein‐ligand interaction; for example in many species binding between oocyte and spermatozoon is orchestrated by O‐linked oligosaccharides found on the zona pellucida protein 3 (ZP3) in the fertilization event.^11^ Also, O‐glycosylation provides a protecting barrier over epithelial surfaces against chemical, physical, and microbial agents, protects from the proteolytic cleavage,^12^ increases the stability of the protein^13^, ^14^ even with the shortest O‐GalNAc on interleukin‐2.^7^ In disease states, alterations of O‐glycans on the cell surface occur which enable cancer cells to be differentiated, and make surface O‐glycans biomarkers in directed therapeutic approaches.^15^, ^16^

However, there is a lack of reliable information on the structure of glycosylated systems for several reasons. Most of the experimentally solved proteins are recombinantly expressed in bacteria such as *E.coli* lacking the glycosylation machinery. Furthermore, biomolecular structures have undergone extensive manipulation of oligosaccharides before X‐ray crystallography or NMR spectroscopy because of their inherent flexibility and high degree of coordination with water. Even though 70 % of all proteins are modified by glycans in human cells, only 3 to 4 % of the structures in the PDB carry glycan chains.^17^, ^18^ Since glycans hamper the crystal growth, in eukaryotic expression systems these units are cleaved, explaining why 80 % of the available X‐ray structures with glycans show only one or two residues of the glycan units. Also, structures from X‐ray crystallography offer the crystalline form of glycosylated systems, lacking a more diverse solution representation. NMR can offer structures in solution; however, these represent averages of simultaneously occurring conformers, limiting the amount of structural information. The complexity of the glycosylation creates obstacles while studying them experimentally. A computational approach opens the way by offering a platform which can be controlled; therefore, a direct reasoning on the effect of a model system can be made. Among the computational methods, molecular dynamics simulation emerges as a powerful tool for the modeling of glycosylated systems by offering a detailed spatial and temporal resolution.

In this context, there is an ongoing effort in developing carbohydrate force fields within different force‐field families. Examples are CHARMM,^19^ GLYCAM06,^20^ GROMOS 53A6‐GLYC,^21^, ^22^ GROMOS 56A6CARBO,^23^, ^24^ MARTINI^25^ (coarse‐grained), and a recent polarazible DRUDE forcefield for carbohydrates.^26^ A review of some these force fields can be found in ref. [27]. Recently, Corzana et al.^28^, ^29^, ^30^ studied O‐glycosylated dipeptides through combined NMR and with NMR restrained MD simulations using the AMBER force field. Also, Mallajosyula et al.^31^ studied 14 glycopeptides (without unglycosylated forms) with Hamiltonian replica exchange (HREX) to validate the CHARMM carbohydrate force field. They used 2D biasing potentials^32^ which have been previously studied in the content of peptidic^33^, ^34^ and oligosaccharide systems.^35^ The GROMOS force field for carbohydrates 53A6GLYC was recently validated for N‐glycans and cyclodextrin.^36^, ^37^

In this work, unbiased MD and local elevation with umbrella sampling (LEUS) simulations have been applied on four glycopeptides which are models of the most common mucin type O‐linkages alongwith their unglycosylated forms. Since the exact effects of these glycans on peptides are unclear, establishment of a reliable computational setup can help to shed light on the resulting structural ensembles. Therefore, we first focused on the more studied systems where there is enough information to compare and validate. To test our force field parameters, the NMR spectroscopy studies of the *α*‐GalNAc‐Ser/Thr and *β*‐GalNAc‐Ser/Thr glycopeptides from ref. [28, 29] were used as reference values.

## 2 Methods

### 2.1 MD Simulation Settings

**Figure 1:**
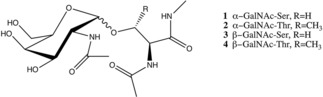
Graphical representation of the glycopeptides used in this study. The ends of the peptide part are patched with N‐acetyl and N‐methyl groups at the N‐ and C‐terminus, respectively in order to be compatible with the experimental composition.

In this study, we have focused on the most common O‐glycosylation type by using the four model systems represented in Figure 1. Systems 1 and 2 are also known as Tn antigen. All MD simulations were performed using the GROMOS11 biomolecular simulation package (http://www.gromos.net)^38^ and the 54A8 GROMOS force field.^39^ For compatibility with the protein force field minor modifications to the original 53A6glyc carbohydrate parameter set were applied.^36^, ^40^ Initial structures of the studied units were modeled in the molecular operating environment (MOE)^41^ by setting their glycosidic dihedral angles to their respective free‐energy minima, which have been previously reported.^36^ A short energy minimization was applied using the steepest descent algorithm. The compounds were placed in a periodic cubic water box with simple point charge (SPC) water^42^ molecules and initialized with a 1.4 nm minimum distance of the solute to the box walls. With position restraints on the solute atoms, the system was further relaxed by a steepest descent minimization before the production run. Then, the systems were equilibrated with initial random velocities generated from a Maxwell‐Boltzmann distribution at 60 K then heated up to 300 K in five discrete steps. While heating up the system, position restraints on the solute atoms were reduced from 2.5×10^4^ to 0.0 kJ mol^−1^ nm^−2^.

The production simulations were performed at a constant temperature of 300 K and a constant pressure of 1 atm using a weak coupling scheme^43^ for both temperature and pressure with coupling times *τ_T_*=0.1 ps and *τ_P_*=0.5 ps, respectively with an isothermal compressibility of 4.575×10^−4^ kJ^−1^ mol nm^3^. Newton's equations of motion were integrated using the leapfrog scheme^44^ with a time step of 2 fs. The SHAKE algorithm^45^ was used to maintain the bond distances at their optimal values. Long‐range electrostatic interactions beyond a cutoff of 1.4 nm were truncated and approximated by a generalized reaction field^46^ with a relative dielectric permittivity of 61.^47^ Nonbonded interactions up to a distance of 0.8 nm, were computed at every time step using a pairlist^48^ that was updated every 5 steps. Interactions up to 1.4 nm, were computed at pairlist updates and kept constant in between.

The GROMOS++ software^49^ is used for time series analysis. A geometrical criterion was used to identify hydrogen bonds if a hydrogen‐acceptor distance is smaller than 0.25 nm and the donor‐hydrogen‐acceptor angle is larger than 135°. Secondary structure propensities are determined as described in Ref. [50], using the definitions in Table S4 in SI.

### 2.2 Parametrization

The backbone parameters of nonglycosylated threonine were reparametrized to better reproduce the experimental J‐values and secondary structure propensities (*α*, *β* and P_II_) from Grdadolnik et al.^51^ A Monte Carlo search scheme was used in combination with Hamiltonian reweighing to identify new dihedral backbone parameters with the closest agreement with experiment.^50^ The reason to parametrization specifically the Thr backbone parameters was the strong dependence of the J‐value on the backbone length and the secondary structure, which was not seen for Ser (Table S3, in SI). Updated parameters for the 54A8 GROMOS force field can be found in Table S3.

### 2.3 Creating Biased Potentials with LE and Sampling with US

For each system, unbiased MD simulations were carried out for 100 ns after equilibration. In addition to unbiased simulations, an enhanced sampling method, local elevation with umbrella sampling (LEUS)^52^, ^53^ was applied. To ensure a near‐to‐complete sampling along both O‐glycosidic linkage and peptide backbone, two 2D LE potentials are built separately for the O‐glycosidic linkage (*φ_S_*, *ψ_S_*) with t_LE_=100 ns and for the peptide backbone (*φ_P_*, *ψ_P_*) with t_LE_=10 ns. In the US phase, the LE biased potentials were frozen and sampling was applied by using both 2D potentials by saving trajectories every 0.1 ps for 100 ns to achieve statistical efficiency as discussed in ref. [52]. In the LEUS method dihedral angles are binned in N_g_=36 bins, a biasing potential width of *σ*=360°/N_g_ was used with a force constant increment of c=0.005 kJ mol^−1^.

The unbiased probability of any property *Q* can be obtained from the LEUS (biased) simulations through reweighing:

(1) $$P\left(Q{}^{\circ }\right)=\frac{\left\langle \delta \left(Q-Q{}^{\circ }\right)\exp\left[U_{\mathrm{LEUS}}\left(Q\right)/k_{B}T\right]\right\rangle }{\left\langle \exp[U_{\mathrm{LEUS}}\left(Q\right)/k_{B}T]\right\rangle }$$

where 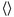 indicates an ensemble average of the biased LEUS simulation, *U*_LEUS_(Q) is the biasing energy at a particular value of *Q*, *δ* is the Kronecker delta function, *k_B_* is the Boltzmann constant and *T* is the absolute temperature. The corresponding free energies can be obtained from the calculated probabilities,

(2) $$G\left(Q\right)=-k_{B}T\ln P\left(Q\right)$$

For glycosylated systems, two free energy maps, G(*φ_S_*, *ψ_S_*) for the glycosidic linkage and G(*φ_P_*, *ψ_P_*) for the protein backbone were created from the LEUS simulations after reweighing of the biased energy with eqs. 1 and 2. To compare the effect of glycosylation, free‐energy maps G(*φ_P_*, *ψ_P_*) of the unglycosylated systems were also created. The global minimum of each map represents the lowest free energy with the highest probability of the state which is set to 0 kJ/mol and the colormap is drawn using a 5 kJ/mol contour. Detailed explanation for the construction of the free‐energy maps can be found in Ref. [36].

### 2.4 ^3^J‐coupling Constants and NOE Calculations

Simulations are compared with NOE data and ^3^J‐coupling constants. Aliphatic carbons atoms are treated as united atoms in the GROMOS force field. Therefore, virtual atomic positions for prochiral CH_2_ (C*β* in Ser), for CH (C*α* and C*β* in Thr) and pseudo atomic positions for CH_3_ (C1 in GalNAc and C*γ* in Thr) were used to calculate interproton distances. For the NOE analysis, since experimental NOE distances represent an average over space and time, and for small molecules the NOE intensity is proportional to *r* ^−6^, averaging is performed as ${\left\langle r^{-6}\right\rangle }^{-1/6}$ .^54^ More elaborate approaches to compute the NMR spectra explicitly from simulation trajectories were shown to deviate by at most 8–9 % from this inverse sixth power assumption.^55^, ^56^

^3^J‐coupling can be related to a torsional angle through the Karplus relation. At particular angles, variations in the Karplus relation parameters (a, b, c) lead to differences of up to 3 Hz even though the experimental uncertainties are generally lower than 0.1 Hz.^57^, ^58^, ^59^

**Figure 2:**
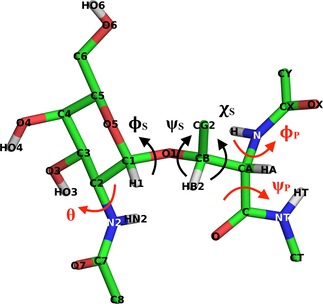
The molecular structure of *α*‐GalNAc‐Thr is represented along with atom names as an example for the rest of the glycopeptides. Dihedral angles are defined as $\phi_{S}$ =O5−C1−O1−C*β*, $\psi_{S}$ =C1−O1−C*β*−C*α*, $\chi_{S}$ =N−C*α*−C*β*−O1, $\phi_{P}$ =CX−N−C*α*−C, $\psi_{P}$ =N−C*α*−C−NT, *θ*=O7−C7−N2−C1.

^3^J_*HNHα*_, ^3^J_*HαHβ*_, and ^3^J_*HNH*2_ coupling constants are related to the dihedral angles of $\phi_{S}$ , $\chi_{S}$ and $\theta_{N-Acetyl}$ (Figure 2), and were calculated from MD and LEUS simulations using the following Karplus relations.^60^, ^61^, ^62^ Note that for Thr there is one ^3^J_*HαHβ*_, while for Ser there are two possible values.

(3) $${\;}^{3}J_{HNH\alpha }=6.51cos^{2}\theta -1.76cos\theta +1.60,\theta \;=\left(\mathrm{CX}-N-C\alpha -C\right)-60{}^{\circ }$$

(4) $${\;}^{3}J_{HNH2}=9.6cos^{2}\theta -1.51cos\theta +0.99,\theta \;=\left(C7-N2-C2-C1\right)+60{}^{\circ }$$

(5) $${\;}^{3}J_{H\alpha H\beta }=9.5cos^{2}\theta -1.6cos\theta +1.80,\theta \;=\left(N-C\alpha -C\beta -O1\right)-120{}^{\circ }\;\mathrm{for}\;\mathrm{Thr}$$

(6) $${\;}^{3}J_{H\alpha H\beta 2}=9.5cos^{2}\theta -1.6cos\theta +1.80,\theta \;=(N-C\alpha -C\beta -O1)\;\mathrm{for}\;\mathrm{Ser}$$

(7) $${\;}^{3}J_{H\alpha H\beta 3}=9.5cos^{2}\theta -1.6cos\theta +1.80,\theta \;=\left(N-C\alpha -C\beta -O1\right)-120{}^{\circ }\;\mathrm{for}\;\mathrm{Ser}$$

where the notation (A−B−C−D) denotes the value of the dihedral angle defined by atoms A, B, C and D.

## 3 Results and Discussion

Unbiased MD and LEUS simulations were analyzed in terms of average NOE‐derived atom‐atom distances, ^3^J_*HNHα*_, ^3^J_*HαHβ*_, and ^3^J_*HNH*2_ couplings, intramolecular hydrogen bonding occurrences, peptide backbone and sugar glycosidic angle distributions.

**Figure 3:**
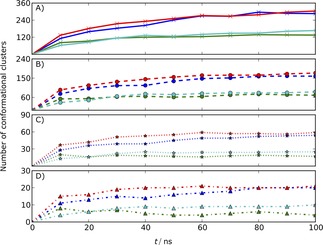
Time course of the number of conformational clusters of studied systems 1: *α*‐D‐GalNAc‐Ser(red), 2: *α*‐D‐GalNAc‐Thr (blue), 3: *β*‐D‐GalNAc‐Ser (green) and 4: *β*‐GalNAc‐Thr (cyan) with a combined weight of 99 % (A), 95 % (B), 75 % (C) and 50 % (D). Each point in the curves of the panels represents the total number of conformational clusters that make up 99 %, 95 %, 75 % and 50 % of the trajectory sampled up to the corresponding time point. A levelling of of the curves implies that no new conformations are sampled.

Overall, we think the LEUS simulations are more appropriate to compare to, due to a more complete sampling. Convergence of the LEUS simulations is shown by performing a conformational clustering over time (see Figure 3 ). First, an all atom root‐mean‐square difference (RMSD) matrix was calculated after fitting of atomic coordinates of the backbone atoms of the peptide and the ring atoms of the sugar. Then, a conformational clustering^63^ was performed on a set of 10000 glycopeptide structures taken at 10 ps intervals from the simulation, using a 0.1 nm cutoff to define similar structures.

Figure 3 shows the development over time of the number of clusters that is needed to capture 99 %, 95 %, 75 % and 50 % of the trajectory. Convergence of these curves indicate that no new conformations are sampled anymore. This occurs for the Thr systems (2 and 4) after about 50 ns and for the Ser systems (1 and 3) after about 60 ns, suggesting a reasonably complete sampling of conformational space. Therefore, all the analyses that are calculated from LEUS simulations are shown in the main text. To show the power of the enhanced sampling, the same analysis from the unbiased MD simulations is presented in the supplementary information. Ensemble averages from LEUS simulations were calculated by reweighing to the unbiased ensemble while for unbiased MD simulations they are calculated by averaging over the whole simulation trajectory. Error estimates are reported as the standard deviation over averages obtained from dividing the trajectories in four equally long blocks.

While the secondary structure propensities from unbiased MD simulations capture the LEUS simulations, overall substantial differences are seen in system 2 and system 4. In system 2 unbiased MD simulation shows 0.50 *β*, 0.35 P_II_ propensity while in LEUS simulations populations of these regions were calculated as 0.38 and 0.51, respectively. This might result from the fact that hydrogen bonding between the amide proton of the N‐acetyl group of GalNAc (HN2) and the oxygen of the carbonyl group of C terminus (O) is stabilizing the *β* conformation with 8 % occurrence among the *β* conformation and this H‐bonding was not captured in the P_II_ conformation (data not shown). Therefore, in LEUS simulations the true population might have been captured better by crossing the energetic barrier associated with breaking the H‐bond. Another difference was seen in system 4, with 0.11 *α* and 0.49 P_II_ content in unbiased MD simulations while these were 0.18 and 0.36 in LEUS simulations. The increase in the P_II_ content in unbiased MD can be attributed to the hydrogen bonding between the amide proton at the N‐terminus (H) and ring oxygen (O5) with an occurrence of 15 % in this region, which is not observed in the *β* region. In addition to this, the *α* content of all glycosylated systems were reduced with respect to the unglycosylated peptides except for system 4 where it had 0.11 and 0.18 *α* content in unbiased MD and LEUS simulations, respectively. H‐bonding was observed between the amide proton at the N‐terminus (H) and the ring oxygen (O5), with 13 % occurrence in the P_II_ region, while it is not observed in the *β* region in MD simulations. The increase in the *α* propensity of system 4 can be explained with the stabilization due to HT‐O5 hydrogen bonding, which is not observed in the other systems.

### 3.1 Free‐Energy Landscape

**Figure 4:**
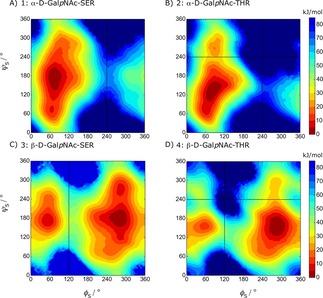
Free‐energy maps G ($\phi_{S}$ , $\psi_{S}$ ) of the glycosidic dihedral angles from the LEUS simulations. Contour maps are drawn with 5 kJ/mol spacing starting from the global minimum energy which is set to 0 kJ/mol. The regions that were never visited are shown in dark blue and the corresponding unbiased free energies are represented in the color maps.

Visual inspection of the glycosidic free‐energy maps (Figure 4) reveals that the *α*‐linked systems (1 and 2) present significant minima for *φ*_S_≈60° (g^+^). In contrast, the *β*‐linked systems (3 and 4) present significant minima for both *φ*_S_≈60° (g^+^) and *φ*_S_≈300° (g^−^) in which the latter one is the lowest energetic state. These observations agree with the exo‐anomeric effect.^64^, ^65^, ^66^

Further investigation of the free‐energy maps reflects the additional steric hindrance upon an additional methyl group in Thr on the conformational preferences of the glycosidic linkage. For *α*‐linked Ser, *ψ*_S_ can span almost all of 0° to 360° when *φ*_S_≈60°; in Thr the additional methyl group splits the energy landscape at *ψ*_S_ ≈240°. This difference between system 1 and 2 has an important implication in biological recognition, since antibodies exhibit different affinities towards glycopeptides having *α*‐linked Ser or *α*‐linked Thr.^67^ Our free‐energy landscapes can capture this difference with the lowest minima at *φ*_S_ ≈60° while *ψ*_S_≈60° and 120° for system 1 and 2, respectively. This was recently confirmed experimentally to be the main conformers in solution and in the bound state.^68^ For *β*‐linked systems, a similar difference is seen for *β*‐linked Thr (system 4) where the upper region with *φ*_S_≈300° and *ψ*_S_≈270° is no longer thermally accessible (higher than 10 k_B_T). In contrast, *β*‐linked Ser exhibits a thermally accessible state at this region.

**Figure 5:**
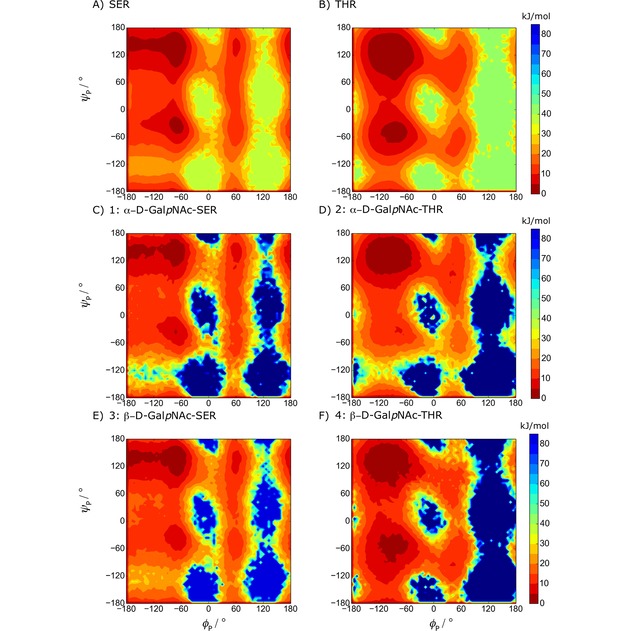
Free‐energy maps G ($\phi_{P}$ , $\psi_{P}$ ) of the protein backbone dihedral angles from the LEUS simulations. Colors are explained in Figure 3.

The free‐energy maps of the protein backbone dihedral for unglycosylated and for the glycosylated systems are shown in Figure 5. One can clearly see the effect of glycosylation on the protein backbone from these free‐energy maps where the $\alpha_{R}$ region becomes an energetically less favorable conformational state, except for system 4.

### 3.2 ^3^J Coupling Constants

#### 3.2.1 ^3^J_HNH*α*_ Couplings

For all the systems 1 to 4 as well as Ser and Thr without glycosylation, Table 1 shows the experimental ^3^J_HNH*α*_ couplings and the computed values from the LEUS simulations (see Table S5 for unbiased MD results). Binned *φ*_P_ preferences are also reported to trace the contribution to the J‐value.

**Table 1:** Experimental and calculated ^3^J_HNH*α*_ coupling constants with propensities from LEUS simulations after reweighing. *φ*_P_ distributions are calculated to track the contribution of each conformation to the J value.

Sys.	Exp.^*a*^	LEUS	LEUS Propensities^*b*^				LEUS *φ* Preferences		
	^3^J_HNH*α*_	^3^J_HNH*α*_	*α*	*β*	P_II_	UC	[−180°, −100°]	[−100°, 0°]	[0°, 180°]
SER	7.0	6.3±0.01	0.17	0.24	0.49	0.10	45.0 %	38.0 %	17.0 %
THR	7.4	7.4±0.01	0.16	0.25	0.44	0.15	37.4 %	48.2 %	14.4 %
1	6.2	6.5±0.2	0.11	0.31	0.50	0.08	37.2 %	56.5 %	6.3 %
2	8.8	7.7±0.1	0.02	0.38	0.51	0.09	23.3 %	75.1 %	1.6 %
3	6.6	6.3±0.1	0.07	0.28	0.56	0.09	31.6 %	63.9 %	4.5 %
4	7.4	7.7±0.1	0.18	0.29	0.36	0.17	43.8 %	54.7 %	1.5 %

^*a*^ Experimental values for Ser & Thr from Ref. [69] and rest are from [28,29], uncertainties not supplied; ^*b*^ Propensities are computed according to the definitions in Figure S2. UC stands for unclassified.

Since the ^3^J_HNH*α*_ coupling constant reflects only the *φ*_P_ torsion angle, one can not distinguish secondary structure preferences. Therefore, propensities were calculated and reported for all the systems in Table 1 for LEUS simulations and in Table S5 for unbiased MD simulations.

First, we investigated the Ser and Thr aminoacids with an acetyl group at the N‐terminus and a N‐methyl group at the C‐terminus to check the protein backbone model without glycosylation. Experimental propensities from Grdadolnik et al.,^51^ obtained from fitting ATR‐Absorbance and Raman data, give exceedingly low populations of alpha conformations with 3 % for Ser and 4 % for Thr, compared to studies from random coil analyses in whole proteins. The reason of the low *α* content is the fact that the system which consists of single amino acid can not form hydrogen bonding; therefore, can not form helical shapes. The sensitivity of the ^3^J_HNH*α*_ value of Thr can also be evidenced with the different experimental studies displaying variation according to length of the backbone; 7.4 Hz in the shortest peptide, 7.9 Hz in the pentapeptide (Table S3). In preliminary simulations of the smallest peptides, it turned out that in particular, the conformational preferences of Thr and its ^3^J_HNH*α*_ couplings did not agree with the available dipeptide experimental data^51^, ^69^ (Table S1). The calculated J coupling constant with the original backbone parameters had a 1.1 Hz deviation from the experimental value, which was greater than the deviation of Ser with 0.7 Hz. This deviation in Thr can be explained by looking at the calculated propensities where it prefers mostly an *α* conformation instead of *β* with the 54A8 parameter set. For this reason, new backbone torsional parameters for Thr are introduced and these parameters are used for *α*/*β*‐GalNAc‐Thr (system 2 and 4). 54A8 and updated backbone dihedral angle parameters (k, $\theta {}^{\circ }$ and m) are presented in Table S2. Altering the backbone dihedral angle parameters in threonine shifted the propensities towards the experimental target values with an increase in *β* population from 0.09 to 0.24. Although the experimental *β* propensity has still not been met (0.58), the deviation in the ^3^J_HNH*α*_ coupling was reduced to 0.01 Hz.

**Figure 6:**
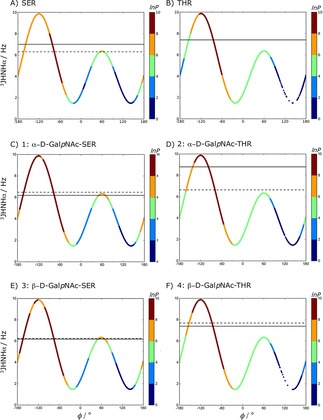
*φ*_P_ vs. calculated ^3^J_HNH*α*_ couplings from LEUS simulations for all studied systems. Experimental and calculated ^3^J values are represented with solid and dashed horizontal lines, respectively. The colors on this Karplus curve indicate the preferred sampling after unbiasing of the LEUS simulations. In the unbiasing procedure, LEUS occurrences (P) are binned with 6° grid spacing. Negative values of *lnP* set to zero.

Next, we turn our attention to the glycosylated systems. A maximum deviation between the computed and measured ^3^J_HNH*α*_‐coupling values of 1.1 Hz was observed for system 1. The rest of the ^3^J_HNH*α*_ couplings for the glycosylated systems were in agreement with the experimental values with a deviation of 0.3 Hz. Compared to system 1, system 2 has a large experimental ^3^J_HNH*α*_‐coupling of 8.8 Hz^28^ which has also been reported by other groups in the 8.3–9.2 Hz range. Coltart et al.^16^ report a value of 6.7–7.0 Hz for system 1 which is still smaller than the values reported for system 2. This increase in ^3^J_HNH*α*_‐coupling of *α*‐linked O‐glycosylation in threonine as compared to serine does not seem to be completely captured in our simulations, even though we do see a pronounced reduction of the *α*‐conformation towards the *β*‐conformations (Table 1) for this system (see also Figure 5). In Figure 6, we visualize where the large deviation in the ^3^J_HNH*α*_‐value comes from. In contrast to the Ser cases, the glycosylated Thr does not sample values of *φ*_P_ in the range [−180°, −120°] as much, and as a result, the ^3^J_HNH*α*_ value does not increase to the extent observed in the experiments. The systems with *β*‐link experimentally show a smaller effect of glycosylation, which we seem to represent more accurately.

**Figure 7:**
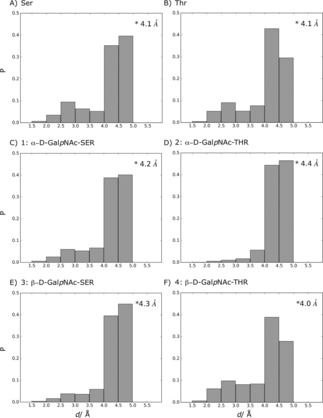
Calculated H_T_,H distance (Å) distributions from LEUS simulations after unbiasing. Overall average unbiased distance of each system is represented in the upper‐right corner of every panel. NMR studies reported that extended backbone conformation shows d(H_T_,H) at 4.4 Å and 4.3 Å for *β* and P_II_, respectively.^70^

Evidence for the extended conformation of system 4 was further checked with the analysis of the HT−H distance (d(H_T_,H)) which was shown to be at 4.4 Å through NMR studies.^70^ For all systems d(H_T_,H) distributions were calculated from LEUS simulations and reported as populations in Figure 7. Upon glycosylation the average value of d(H_T_,H) increases, except for system 4 which exhibits a decrease due to an increase in the population of the bins from 1.5 Å to 3.5 Å.

The notable preference for *α*‐helical conformations at the expense of more extended P_II_ conformations may offer an explanation why *β*‐linked GalNAc is not observed in naturally occurring O‐glycosylations, which are mostly observed in extended (P_II_) regions of the protein. There are *β*‐linked O‐glycosylation examples, but they are seen with GlcNAc (N‐acetylglucosamine), Gal (Galactose), Glc (Glucose) or Xyl (Xylose).

#### 3.2.2 ^3^J_*HαHβ*_ Couplings

Table 2 compares multiple measured ^3^J_H*α*H*β*_ values to the computed values from LEUS simulations; for unbiased MD simulation results see Table S6. Note that Ref. [16] measured ^3^J_H*α*H*β*_ in a pentapeptide (STTAV). As this seems to be the most relevant available data for the unglycosylated systems, we also include the corresponding values for the glycosylated systems. For system 4, we see the effect of more complete sampling in LEUS, where the J‐value drops from 6 Hz in plain MD to 3.5 Hz in the LEUS simulations, in agreement with experimental values 3.5 Hz^29^ or 4.6 Hz.^16^ Our simulations reproduce the increase in the experimental J‐value from *α* to *β*‐linked Thr with about 0.5 Hz deviation from the averaged experimental values (*α*‐linked 2.4 Hz; *β*‐linked 4.0 Hz). The population of $\chi_{S}$ that is in the [−120°, 0°] region increases by 9 %. This increase is also depicted in Figure 4, where this region changes its color from green to orange showing an increase in the population. Since this region corresponds to a higher J‐value, increase in this population results in an higher J‐value for system 4 compared to system 2. The same effect is seen for systems 1 and 3 *β*‐linked GalNAc shows an increase from 6.7 to 9.4 Hz when compared to the *α*‐linked analogue. Experimentally, this increase is also observed, albeit considerably less pronounced in Refs. [28, 29] than in [16]. For these systems the increase in ^3^J_H*α*H*β*_ comes from the doubling of the anti population ([−180°, −120°], [120°, 180°]). This region of the Karplus curve corresponds to the steepest and most sensitive edges, resulting in large deviations from a small difference in the dihedral angle, which probably explains the deviation from the experimental values.

**Table 2:** Experimental and calculated ^3^J_H*α*H*β*_ coupling constants from LEUS simulations after reweighing. *χ*_S_ distributions are calculated to track the contribution of each conformation to the J value.

Sys.	Exp.	Exp.	LEUS	LEUS	LEUS *χ*_S_ Preferences		
	^3^J_*HαHβ*_	^3^J_*αHβ2*_	^3^J_*HαHβ*_	^3^J_*HαHβ2*_	[−180°, −120°]	[−120°, 0°]	[0°, 120°]
					[120°, 180°]		
SER	5.8^*a*^	5.9^*a*^	6.8±0.08	5.6±0.04	38.5 %	28.7 %	32.8 %
THR	4.2^*a*^; 5.0^*a*^		3.9±0.01		0.0 %	13.1 %	89.9 %
1	5.5^*b*^; 5.2^*a*^	4.5^*b*^, 3.3^*a*^	6.7±2.0	6.0±0.8	38.7 %	34.1 %	27.2 %
2	2.5^*c*^; 2.3^*a*^		2.8±0.1		7.3 %	0.7 %	92.0 %
3	6.8^*c*^; 8.0^*a*^	–^*c*^, 5.5^*a*^	9.4±1.1	4.7±1.3	66.4 %	19.3 %	14.3 %
4	3.5^*c*^; 4.6^*c*^		3.5±0.5		9.8 %	9.5 %	80.7 %

^*a*^ Experimental values from ref. [16]; ^*b*^ from ref. [28]; ^*c*^ from ref. [29], uncertainties not supplied.

**Figure 8:**
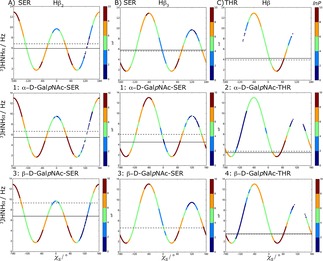
$\chi_{S}$ vs. calculated ^3^J_H*α*H*β*_ coupling from LEUS simulations for all studied systems. Experimental and calculated average ^3^J_H*α*H*β*_ values are represented with solid and dashed horizontal lines, respectively. Only the dipeptide experimental values from Refs. [28, 29] are compared here. The colors indicate the preferred sampling after unbiasing of the LEUS simulations.

Overall, for this J‐value, the Thr systems agree excellently with the measured data, while larger deviations are observed for the Ser systems. From Figure 8 we learn that the average values obtained in the simulations are the result of sampling three conformations with lower and higher J‐values than observed in the experiment. A slight shift in the conformational preferences towards the gauche +/− conformations could improve the agreement. Furthermore, it becomes clear from Figure 8 that the $\chi_{S}$ angle of the Thr systems are more restricted than the Ser systems. While $\chi_{S}$ can take all three conformations with highest populations (represented in red) in the Ser systems, in the Thr systems conformations around +60° are preferred over the other conformations.

#### 3.2.3 ^3^J_*HNH*2_ Couplings

**Figure 9:**
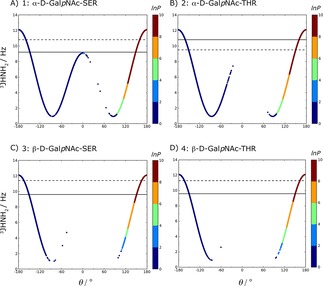
*θ* vs. calculated ^3^J_*HNH*2_ couplings from LEUS simulations after reweighing for glycosylated systems. Experimental and calculated average ^3^J_*HNH*2_ values are represented with solid and dashed horizontal lines, respectively. The colors indicate the preferred sampling after unbiasing of the LEUS simulations.

Finally, we consider the ^3^J_*HNH*2_‐values that result from the N‐acetyl group in GalNAc. The results are summarized in Table 3 and Figure 9 for LEUS simulations and Table S7 for MD simulations. No significant differences between plain MD and LEUS were observed. The simulations seem to consistently overestimate the ^3^J_*HNH*2_‐values. As can be seen in Figure 9, we do sample the correct dihedral angles, but because these regions correspond to the steep areas of the Karplus curve, small deviations in the angle (or the Karplus constants) lead to large deviations in the J‐value. Comparison of the *α* and *β* systems shows that there is a slight increase in the calculated J‐values for *β* systems (0.7 Hz) which can be explained by looking at Figure 9 where the gauche conformations are not accessible in the *β* systems (which corresponds to a lower J‐value) causing an increase in the overall J‐value.

**Table 3:** Experimental and calculated ^3^J_HNH2_ coupling constants from LEUS simulations after reweighing. *θ* distributions are calculated to track the contribution of each conformation to the J value.

Sys.	Exp.^*a*^	LEUS	LEUS Preferences		
	^3^J_HNH2_	^3^J_HNH2_	[−180°, −120°]	[−120°, −60°]	[−60°, 60°]
			[120°, 180°]	[60°, 120°]	
1	9.2	10.7±0.2	98.9 %	1.1 %	0.1 %
2	9.5	10.8±0.1	99.2 %	0.8 %	0.0 %
3	9.6	11.4±0.1	100.0 %	0.0 %	0.0 %
4	9.6	11.3±0.1	99.9 %	0.1 %	0.0 %

^*a*^ Experimental values from ref. [28, 29], uncertainties not supplied.

### 3.3 NOE Analysis

As the systems consist of small molecules for which the molecular tumbling is fast, we used r^−6^ averaging to compare distances observed in the simulations to those derived from NOE intensities. To check this hypothesis, the rotational relaxation time of the studied systems were calculated from the autocorrelation function of the Legendre polynomials. Three vectors were defined; one from the C*α* atom to the center of mass of the sugar moiety, a second one from the peptide backbone atoms N to C, and the last one as the cross product of the two. For each of these vectors, the auto‐correlation function is computed and fitted to an exponential function of the form $Ae^{-k/\tau }+c$ . After fitting, the decay constant was calculated as *τ*=0.5 ns which is at least a factor 10 faster than for typical proteins.

Trajectories from unbiased MD simulations and LEUS simulations after unbiasing were checked with average interatomic distances, see Table 4. Deviations of the calculated averaged distances from the experimental NOE bounds of less than 1 Å are considered as insignificant. All the calculated averaged NOE distances are within 1 Å except for the HT−H distance in system 2 (see Figure S1 for the labeled system). As the HT to HA NOE seems to be fulfilled, the reason may be found in the distribution of the backbone angle. A shift of this angle towards values of −180° may be sufficient to fulfill both the NOE and bring the high ^3^J_HNH*α*_ in closer agreement with experiment (see Table 1 and Figure 6).

**Table 4:** Comparison of experimental NOE data with r^−6^ averaged distances in Å obtained from unbiased MD and LEUS simulations (reweighted) for systems 1–4.

NOE	Exp.^*a*^	MD	MD	LEUS	LEUS
			violation		violation
1 : *α*‐D‐GalNAc‐Ser					
d(H_T_,H)	2.9	3.4±0.02	0.5	3.4±0.2	0.5
d(H_A_,H_T_)	2.3	2.3±0.01	0	2.3±0.06	0
d(H_A_,H)	2.9	2.6±0.02	0	2.7±0.2	0
d(H_T_,H_B1_)	2.5	3.5±0.01	1.0	3.2±0.2	0.7
d(H_T_,H_B2_)	2.6	3.2±0.06	0.6	3.1±0.2	0.5
d(H_1_,H_A_)	3.9	3.8±0.01	0	3.7±0.6	0
2 : *α*‐D‐GalNAc‐Thr					
d(H_T_,H)	2.8	3.8±0.03	1.0	4.0±0.6	1.2
d(H_A_,H_T_)	2.4	2.2±0.3	0	2.2±0.6	0
d(H_A_,H)	2.9	2.8±0.01	0	2.7±0.03	0
d(H_T_,H_B_)	2.8	3.2±0.01	0.4	2.8±0.1	0
d(H,H_B_)	3.5	3.0±0.2	0	3.4±0.4	0
d(H,H_N2_)	3.3	4.0±0.06	0.6	3.4±0.3	0.1
3 : *β*‐D‐GalNAc‐Ser					
d(H_T_,H)	2.9	3.4±0.03	0.5	3.6±0.2	0.7
d(H_A_,H_T_)	2.2	2.3±0.01	0.1	2.3±0.00	0.1
d(H_A_,H)	2.6	2.6±0.04	0	2.7±0.1	0.1
d(H_T_,H_B1_)	2.6	3.4±0.01	0.8	3.4±0.2	0.8
d(H_T_,H_B2_)	2.8	3.3±0.04	0.5	3.4±0.3	0.6
4 : *β*‐D‐GalNAc‐Thr					
d(H_T_,H)	3.0	3.4±0.02	0.4	3.1±0.1	0.1
d(H_A_,H_T_)	2.3	2.3±0.01	0	2.3±0.06	0
d(H_A_,H)	2.8	2.7±0.01	0.1	2.7±0.1	0.1

^*a*^ Experimental values from ref. [28, 29], uncertainties not supplied.

NMR studies of O‐glycosylation resulting in a strong NOE for d(H_A_,H_T_) along with weak NOE for d(H_T_,H) have been suggested to indicate that the peptide backbone is taking an extended conformation.^70^ The distance distributions from LEUS simulation after unbiasing are given in Figure 7. The data in Table 2 confirms further that the major populations are the joint *β* and P_II_ conformations. This is also corroborated by the conformational preferences illustrated in Figure 5. Finally, the increased preference for *α*‐conformations described for system 4 may be reflected by the lack of experimental NOE's for $d(H{,H}_{B})$ , d(H_T_,H_B_) and d(H_T_,H_N_).

### 3.4 Hydrogen Bonding

The occurrence of H‐bonds was reweighed to the unbiased ensemble for the LEUS simulations. H‐bonds with occurrences larger than 2 % are reported in Table 5.

**Table 5:** Hydrogen bond occurrences in LEUS simulations for systems 1–4. Reweighted percentages higher than 2 % are reported.

H‐bond		System			
type		1	2	3	4
sugar‐peptide	HN2−O	4.7 %	7.3 %	–	–
	HN2−OX	5.2 %	6.9 %	–	–
	H−O5	–	–	2.6 %	6.5 %
	HT−O5	–	–	–	5.4 %
peptide‐peptide	HT−OX	2.4 %	–	4.0 %	–
sugar‐sugar	HO4−O6	2.5 %	2.7 %	–	–

Hydrogen bonding between the amide proton from the N‐acetyl group of the GalNAc unit (HN2) and the oxygen atom of carbonyl oxygens (O) or (OX) of Thr and Ser were observed in *α*‐linked systems but not in *β*‐linked systems. This intermolecular hydrogen bonding pattern seems prominent as it was reported by several other groups as well.^16^, ^71^, ^72^ The Ser systems (system 1 & 3) had significantly fewer sugar‐peptide hydrogen bonds compared to systems with Thr (system 2 & 4) which was also observed in the NMR studies of Ref. [28].

For *β*‐linked GalNAc systems, H‐bonds are observed between backbone amide proton (H) or (HT) and ring oxygen (O5). Also for these H‐bonds, a higher occurrence was observed for Thr compared to Ser. Previously in the work of Mallajosyula et al.,^31^ hydrogen bonding in the GalNac systems was observed to be very low (<0.1 propensity) while 0.33 hydrogen bond occurrence was observed in the *β*‐GlcNAc‐Thr system between HO6 (sugar) and O (Thr). Also for others, a similar increase in hydrogen bonding between *β*‐linked sugar and Thr observed in simulations of *β*‐Glucopyranose, *β*‐N‐acetylglucosamine and *β*‐Mannopyranose linked systems.^31^ As an alternative to direct H‐bonding between the sugar and the peptide backbone, water bridged H‐bonds have been suggested.^68^ High occurrences of water bridged H‐bonds between H and O7 or OG and O5 were observed with 8 and 11 % for system 4 while the corresponding direct H‐bonds were not observed or not highly populated.

For the unglycosylated Ser and Thr, the only hydrogen bond (higher than 2 %) was found between the amide proton at the C terminus (HT) and the oxygen at the N‐terminus (OX), with 2.6 and 2.2 %, respectively (i, i+2). HT‐OX was found in systems 1 and 3 among the glycosylated systems with 2.4 and 4.0 %, respectively but not in systems with Thr. In addition to peptide‐peptide H‐bonding, the only significant intrasugar hydrogen bonding was HO4‐O6 which was observed in *α*‐linked systems with 2.5 % and 2.7 % for systems 1 and 2.

Overall, the Thr systems display higher occurrence of H‐bonding which might be an explanation of the superior efficiency of Thr glycosylation. Also, compared to N‐glycosylation where the core‐glycan is sticking out from the peptide backbone, O‐GalNac glycosylation of Thr affects the backbone conformation of the peptide through stronger interactions with the peptide which was also observed in the studies of Ref. [73].

## 4 Conclusions

In this work, we studied O‐glycosylation by glycosylating the single‐residue dipeptides (N‐acetyl‐Ser/Thr‐N‐methylamide) as they represent the simplest system. These model systems allowed us to comment on the direct effect of the glycosylation by eliminating the neighbouring residue effect on the backbone conformation.

By using LEUS as an enhanced sampling method it was possible to cover most of the energetically possible states. We have first investigated unglycosylated systems and reparametrized them according to the available experimental data with J‐couplings and secondary structure propensities. Subsequently the four model systems with *α* and *β*‐linked O‐glycosylation were compared to the NMR experimental findings of the same molecules. We conclude that the current forcefield parameters represent the J‐values and NOE's without introducing any restrains. For ^3^J_HNH*α*_ couplings, the biggest deviation was seen in system 2 with 1.1 Hz while the rest were about 0.3 Hz. For ^3^J_H*α*H*β*_ couplings an increase in the population of the anti region in system 3 created a big deviation with 2.6 Hz where the Karplus curve is steep causing a significant change from a small difference in the dihedral angle.

Alternative experiments showed large variation in exactly this ^3^J‐coupling, reducing the deviation with our calculations to 1.2 Hz. Furthermore, the trends in the ^3^J‐values between systems were captured quite well. For the NOE's the only significant violation was seen in system 2 with 1.2 Å corresponding to the slight deviation in the ^3^J_HNH*α*_ described above. From the propensities that we have calculated for each system we conclude that O‐glycosylation seems to drive the peptide backbone to an extended conformation, with the exception of *β*‐GalNAc‐Thr where the *α*‐helical propensity remains relatively high. In general, Thr systems engage in a closer interaction with the peptide backbone by forming more stable hydrogen bonds. *β*‐GalNAc‐Thr displayed an overall shorter d(H_T_,H) distance than the unglycosylated Thr and an increased preference for *α*‐helical conformations compared to the other glycosylated systems. These slight shifts towards *α*‐helical conformations may be sufficient to explain the evolutionary preference of *α*‐linked GalNAc over *β*‐linked GalNAc.

Overall, we conclude that the GROMOS force field describes the O‐glycosylated systems very well in relation to the available experimental data from NMR.

## Conflict of interest

The authors declare no conflict of interest.

## Supporting information

As a service to our authors and readers, this journal provides supporting information supplied by the authors. Such materials are peer reviewed and may be re‐organized for online delivery, but are not copy‐edited or typeset. Technical support issues arising from supporting information (other than missing files) should be addressed to the authors.

Supplementary
