# Supplementary material for: Structural Aspects of the O‐glycosylation Linkage in Glycopeptides via MD Simulations and Comparison with NMR Experiments
Source: Chemphyschem. 2019 May 6;20(11):1527–37. doi: 10.1002/cphc.201900079 (PMC6563056; doi:10.1002/cphc.201900079)
Supplement: Supplementary file 1 — Supplementary [file CPHC-20-1527-s001.pdf]

CHEMPHYSCHEM

Supporting Information

© Copyright Wiley-VCH Verlag GmbH & Co. KGaA, 69451 Weinheim, 2019

Structural Aspects of the O-glycosylation Linkage in Glycopeptides via MD Simulations and Comparison with NMR Experiments

Aysegül Turupcu, Matthias Diem, Lorna J. Smith, and Chris Oostenbrink*© 2019 The Authors. Published by Wiley-VCH Verlag GmbH & Co. KGaA.
This is an open access article under the terms of the Creative Commons Attribution License, which permits use, distribution and reproduction in any medium, provided the original work is properly cited.

Table S1: Basin definitions used for the calculation of the propensity taken from [1]. See Figure S2 for a graphical representation.

type	ϕ_1°	ϕ_2°	ψ_1°	ψ_2°
α_R	-95	-40	-70	-32
	107	-40	-32	-12
	165	-95	-70	-32
	107	-40	-12	8
	150	-67	8	40
	150	-107	-32	8
β	-135	-100	95	150
	-175	-135	95	136
	-180	-135	136	180
	-135	-105	150	180
	-180	-135	-180	-160
α_L	38	140	-25	75
P_II	-100	-30	95	180
	-100	-60	-180	-150

Table S2: Experimental $^3J_{HNH\alpha}$ values from different studies for Ser and Thr. The J coupling of the Thr which corresponds to ϕ, displays variation among the experimental results indicating the sensitivity of the property to the subtle variation in the length of the protein backbone and the size of the system.

Sys.	Ac-X-NHMe[a]	Ac-X-NHMe[b]	Ac-GGXGG-NHMe[c]	PDB[d]
SER	7.0	7.0	7.0	6.9
THR	7.4	7.3	7.9	7.7

[a] Experimental values at pH 4.9 and T = 30° taken from ref. [2] and [b] at pH 2.9; [c] at pH 5.0 and T = 20° from ref. [3]; [d] from ref. [4]

Table S3: Updated forcefield parameters for ϕ, ψ along with the original 54A8 force field parameters are represented where k is force field constant (kJ/mol), $\theta°$ is phase shift (deg) and m is the multiplicity

	54A8 force field			This study[a]		
	k	$\theta°$	m	k	$\theta°$	m
ϕ	2.8	0	3	1.21	180	1
	0.7	180	6			
ψ	3.5	180	2	1.51	0	1
	0.4	0	6			

[a] Recently we introduced separate terms for groups of amino acids Gly, Ala, common and β-branched amino acids. [1] Here, the parameters are only optimized for single Thr as described in the Methods part. For general purposes, we recommend the use of the β-branched amino acids.

Table S4: Experimental and calculated $^3J_{HNH\alpha}$ coupling constants with propensities from unbiased MD simulations. ϕ_P distributions are calculated to track the contribution of each conformation to the J value.

Sys.	Exp.[a]	MD	MD Propensities[b]				MD ϕ_P Preferences		
	$^3J_{HNH\alpha}$	$^3J_{HNH\alpha}$	α	β	P_{II}	UC	[-180°,-100°]	[-100°,0°]	[0°,180°]
SER	7.0	6.3±0.03	0.15	0.24	0.50	0.10	29.4%	65.4%	5.2%
THR	7.4	7.4±0.04	0.16	0.24	0.46	0.14	33.9%	65.2%	0.9%
1	6.2	6.5±0.03	0.09	0.34	0.44	0.12	39.1%	54.3%	6.6%
2	8.8	7.9±0.1	0.02	0.50	0.35	0.13	28.4%	67.9%	3.7%
3	6.6	6.4±0.04	0.08	0.31	0.48	0.13	36.2%	56.3%	7.5%
4	7.4	7.1±0.05	0.11	0.23	0.49	0.17	32.5%	66.1%	1.4%

[a] Experimental values taken from ref. [2]
[b] Propensities are computed according to the definitions in Figure S2. UC stands for unclassified.

Table S5: Comparison of the experimental and calculated $^3J_{HNH\alpha}$ coupling constants (associated with ϕ_P) and propensities for Ser and Thr amino acids.

Sys.	Exp.[a] $^3J_{HNH\alpha}$	MD 54A8	Exp. Propensities[b] α	β	P_{II}	MD Propensities[c] α	β	P_{II}	UC
SER	7.0	6.3±0.03	0.04	0.47	0.49	0.15	0.24	0.50	0.11
THR	7.4	7.4±0.04	0.03	0.58	0.39	0.16	0.24	0.46	0.14
THR*		6.3±0.04				0.40	0.09	0.45	0.06

* Thr with unmodified parameters

[a] Experimental values taken from ref.[2]

[b] Experimental propensities taken from [3]

[c] Propensities computed using the definitions in Figure S2. UC stands for unclassified conformations.

Table S6: Experimental and calculated $^3J_{H\alpha H\beta}$ coupling constants from unbiased MD simulations. χ distributions are calculated to track the contribution of each conformation to the J value.

Sys.	Exp. $^3J_{H\alpha H\beta}$	Exp. $^3J_{H\alpha H\beta 2}$	MD $^3J_{H\alpha H\beta}$	MD χ_S Preferences [-180°, -120°] [120°, 180°]	[-120°, 0°]	[0°, 120°]
SER	5.8[a]	5.9[a]	6.8±0.3	38.4%	31.9%	29.7%
THR	4.2[a]; 5.0[a]		3.9±0.2	0.0%	12.6%	87.4%
1	5.5[b]; 5.2[a]	4.5[b], 3.3[a]	7.8±1.0	54.0%	32.9%	13.1%
2	2.5[c]; 2.3[a]		2.9±0.05	63.1%	1.4%	35.5%
3	6.8[c]; 8.0[a]	-[c], 5.5[a]	8.9±0.04	60.1%	20.3%	19.6%
4	3.5[c]; 4.6[c]		6.0±0.5	0.0%	37.0%	63.0%

[a] Experimental values from ref. [5]; [b] from ref.[6]; [c] from ref. [7]

Table S7: $^3J_{HNH_2}$ coupling constants from unbiased MD simulations presented with the compared experimental values. Distributions are calculated to track the contribution of each conformation to the J value.

Sys.	Exp.[a] $^3J_{HNH_2}$	MD $^3J_{HNH_2}$	MD Preferences [-180°,-120°] [120°,180°]	[-120°,-60°] [60°,120°]	[-60°,60°]
1	9.2	10.9±0.04	98.7%	0.8%	0.5%
2	9.5	10.8±0.03	98.2%	0.8%	1.0%
3	9.6	11.3±0.03	99.4%	0.1%	0.5%
4	9.6	11.3±0.02	99.9%	0.1%	0.0%

[a] Experimental values from ref.[6, 7]

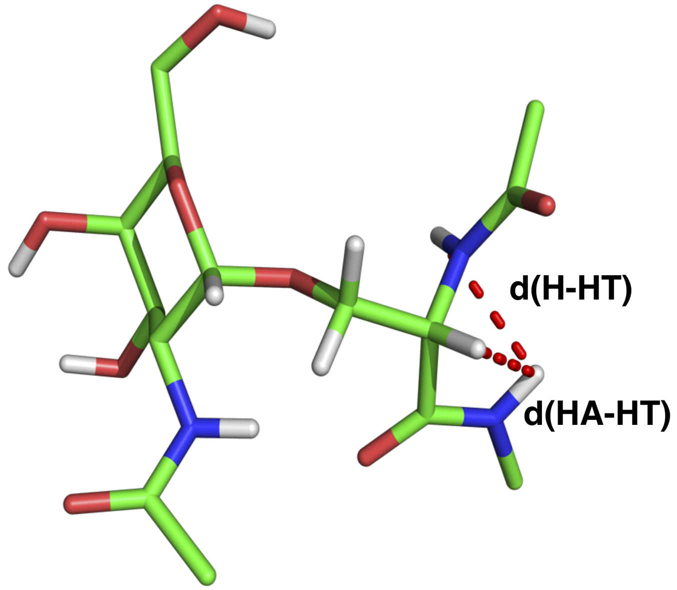

Figure S1: Representation of the distances d(H_A,H_T) and d(H_T,H) for system 1. These distances corresponds to NOE's used in identifying extended conformation of the peptide backbone.

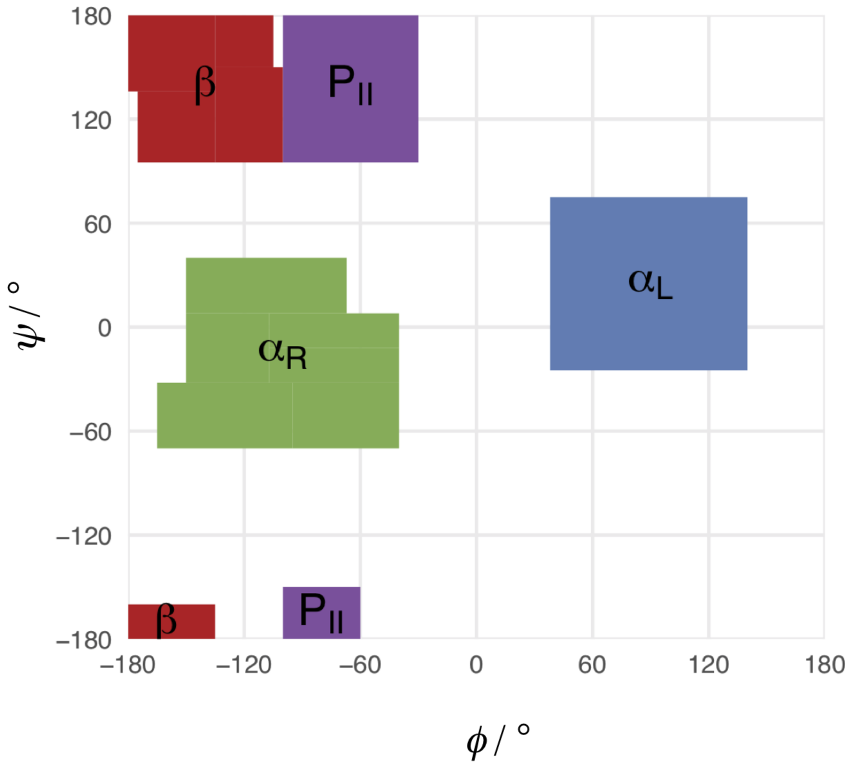

Figure S2: Representation of the backbone dihedral angle subspace with the colored regions corresponding to amino acid propensity definitions adapted from ref. [1]

References

[1] C. Margreitter, C. Oostenbrink, *J. Chem. Inf. Model.* **2016**, *56*, 1823–1834.

[2] F. Avbelj, S. G. Grdadolnik, J. Grdadolnik, R. L. Baldwin, *Proc. Natl. Acad. Sci. U. S. A.* **2006**, *103*, 1272–7.

[3] J. Grdadolnik, V. Mohacek-Grosev, R. L. Baldwin, F. Avbelj, *Proc. Natl. Acad. Sci. U. S. A.* **2011**, *108*, 1794–8.

[4] Y. Mimura, Y. Inoue, N. J. Maeji, R. Chûjô, *Int. J. Pept. Protein Res.* **1989**, *34*, 363–8.

[5] D. M. Coltart, A. K. Royyuru, L. J. Williams, P. W. Glunz, D. Sames, S. D. Kuduk, J. B. Schwarz, X.-T. Chen, S. J. Danishefsky, D. H. Live, *J. Am. Chem. Soc.* **2002**, *124*, 9833–9844.

[6] F. Corzana, J. H. Busto, G. Jiménez-Osés, J. L. Asensio, J. Jiménez-Barbero, J. M. Peregrina, A. Avenoza, *J. Am. Chem. Soc.* **2006**, *128*, 14640–14648.

[7] F. Corzana, J. H. Busto, G. Jiménez-Osés, M. García de Luis, J. L. Asensio, J. Jiménez-Barbero, J. M. Peregrina, A. Avenoza, *J. Am. Chem. Soc.* **2007**, *129*, 9458–9467.
